# PSMA PET/CT imaging for biochemical recurrence in prostate cancer: can it replace conventional imaging and guide salvage therapy?

**DOI:** 10.1007/s12149-026-02192-2

**Published:** 2026-04-13

**Authors:** Efrah Ahmed Ibrahim

**Affiliations:** Department of Nuclear Medicine, Bursa Uludag University, Bursa, Türkiye

**Keywords:** Prostate cancer, Biochemical recurrence, PSMA PET/CT, Conventional imaging, Salvage therapy, Detection rate, Sensitivity, Specificity, Metastasis, Treatment planning

## Abstract

Biochemical recurrence (BCR) after primary treatment for prostate cancer presents a significant clinical challenge. Conventional imaging modalities have limited sensitivity for detecting sites of recurrence, particularly at low prostate specific antigen (PSA) levels. Prostate specific membrane antigen positron emission tomography/computed tomography (PSMA PET/CT) has emerged as a promising diagnostic tool that may transform the management paradigm for recurrent prostate cancer. To systematically evaluate the diagnostic performance of PSMA PET/CT in patients with biochemical recurrence of prostate cancer, compare it with conventional imaging modalities, and assess its impact on salvage therapy decisions. A systematic review was conducted following the PRISMA guidelines. PubMed/MEDLINE, Embase, Cochrane Library, and Web of Science were searched for studies published between 2020 and 2025. Studies evaluating PSMA PET/CT in patients with biochemical recurrence after primary treatment were included. The primary outcomes were detection rates stratified by PSA levels, diagnostic accuracy compared to conventional imaging, and the impact on management decisions. PSMA PET/CT demonstrated superior detection rates compared to conventional imaging across all PSA levels, with detection rates ranging from 36.2% to 96.7% for PSA levels below 0.5 ng/mL and above 5 ng/mL, respectively. In patients with negative conventional imaging, PSMA PET/CT identified true positive lesions in 64% of cases (95% CI, 56.7%-71.5%). Among patients with high-risk biochemically recurrent prostate cancer and negative conventional imaging, PSMA PET/CT detected distant metastatic disease (M1) in 46% of patients. Management plans were altered in 54-76% of cases based on PSMA PET/CT findings, with significant implications for salvage therapy targeting. PSMA PET/CT significantly outperforms conventional imaging in detecting sites of recurrence across all PSA levels, with particularly notable advantages at low PSA values. The high detection rates and accuracy of PSMA PET/CT in patients with negative conventional imaging support its role as a replacement for or complement to standard imaging protocols, while acknowledging current limitations in availability, cost, and long-term outcome data. PSMA PET/CT findings frequently alter management decisions, enabling more precise targeting of salvage therapies and potentially improving oncological outcomes.

**Supplementary Information:**

The online version contains supplementary material available at 10.1007/s12149-026-02192-2.

## Introduction

Prostate cancer remains one of the most common malignancies in men worldwide, with a significant proportion of patients experiencing biochemical recurrence (BCR) after primary treatment [1]. BCR is defined as a rise in prostate-specific antigen (PSA) levels to ≥0.2 ng/mL following radical prostatectomy or an increase of 2 ng/mL above the nadir value after definitive radiotherapy [2]. The management of BCR presents a significant clinical challenge, as it necessitates accurate identification of the site and extent of recurrence to guide appropriate salvage therapy decisions.

Conventional imaging modalities, including computed tomography (CT), magnetic resonance imaging (MRI), and bone scintigraphy, have traditionally been used for restaging patients with BCR. However, these techniques have demonstrated limited sensitivity, particularly at low PSA levels (<1.0 ng/mL), where early detection would be most beneficial for successful salvage treatment [3]. This diagnostic gap has led to a significant clinical dilemma: how to optimally manage patients with rising PSA levels but negative conventional imaging results.

The advent of molecular imaging targeting the prostate-specific membrane antigen (PSMA) has revolutionized the diagnostic landscape for recurrent prostate cancer. PSMA is a transmembrane glycoprotein that is overexpressed in most prostate cancer cells, with expression levels correlating with tumor aggressiveness, metastatic disease, and castration resistance [4]. PSMA positron emission tomography/computed tomography (PSMA PET/CT) combines the high sensitivity of PET with the anatomical precision of CT, enabling detection of small lesions with high contrast resolution.

Several PSMA-targeting radiotracers have been developed, including 68Ga-PSMA-11, 18F-PSMA-1007, and 18F-DCFPyL (also known as 18F-flotufolastat), each with distinct pharmacokinetic properties that influence their diagnostic performance [5]. The superior detection rates of PSMA PET/CT compared to conventional imaging have been consistently demonstrated across multiple studies, with detection rates ranging from approximately 40% at PSA levels <0.5 ng/mL to over 90% at PSA levels >2 ng/mL [6].

The clinical implications of PSMA PET/CT findings are profound, as they frequently lead to changes in management plans, particularly regarding the decision between local salvage therapy and systemic treatment approaches. The ability to detect previously occult metastatic disease in patients with negative conventional imaging has challenged traditional treatment paradigms and raised important questions about the optimal integration of PSMA PET/CT into clinical practice [7].

Despite the growing body of evidence supporting the superior diagnostic performance of PSMA PET/CT, several important questions remain. Can PSMA PET/CT definitively replace conventional imaging in the workup of patients with BCR? How should PSMA PET/CT findings guide salvage therapy decisions? Does PSMA PET/CT-directed therapy lead to improved oncological outcomes? What is the cost-effectiveness of incorporating PSMA PET/CT into standard diagnostic algorithms?

This systematic review aims to comprehensively evaluate the current evidence regarding the diagnostic performance of PSMA PET/CT in patients with BCR, compare it with conventional imaging modalities, and assess its impact on salvage therapy decisions. By combing the latest research findings, including recent phase 3 trials and large cohort studies, we seek to provide evidence-based recommendations for the optimal integration of PSMA PET/CT into the management pathway for patients with recurrent prostate cancer.

## Methods

### Search strategy and study selection

Published systemic reviews/meta-analysis were used to contextualize and support the author narrative. This systematic review was conducted following the Preferred Reporting Items for Systematic Reviews and Meta-Analyses (PRISMA) guidelines. A comprehensive literature search was performed using PubMed/MEDLINE, Embase, Cochrane Library, and Web of Science databases for studies published between January 2020 and March 2025. The search strategy combined terms related to "PSMA PET/CT," "biochemical recurrence," "prostate cancer," "conventional imaging," and "salvage therapy" (a detailed search strategy is available in the Supplementary Material). The articles were subsequently assessed based on predefined inclusion and exclusion criteria.

### Inclusion and exclusion criteria

Studies were included if they met the following criteria:

(1) evaluated PSMA PET/CT in patients with biochemical recurrence after primary treatment for prostate cancer; (2) included a minimum of 20 patients; (3) reported detection rates, sensitivity, specificity, or impact on management; (4) were published in English; and (5) were peer-reviewed original research articles, including prospective or retrospective studies, systematic reviews, or meta-analyses.

Exclusion criteria were: (1) studies focusing solely on primary staging of prostate cancer; (2) studies evaluating only non-PSMA radiotracers; (3) case reports and small case series (<20 patients); (4) conference abstracts without full-text publication; (5) animal or in vitro studies; (6) duplicate publications or overlapping patient cohorts; and (7) studies without clear reporting of methods or results.

### Data extraction and quality assessment

Data extraction was performed independently by the author (E.A.I.) using a standardized form. Uncertainties regarding data extraction were resolved through consultation with a senior supervisor (Prof. Eray Alper). The following information was collected: study characteristics (author, year, country, design), patient characteristics (number, age, PSA levels, prior treatments), PSMA PET/CT protocol details (radiotracer, activity, acquisition parameters), conventional imaging details (when applicable), detection rates overall and stratified by PSA levels, diagnostic performance metrics (sensitivity, specificity, PPV, NPV), impact on management decisions, and outcomes of salvage therapy guided by PSMA PET/CT findings.

The quality of the included studies was assessed using the Quality Assessment of Diagnostic Accuracy Studies-2 (QUADAS-2) tool for diagnostic accuracy studies, the Newcastle-Ottawa Scale for observational studies, the Cochrane Risk of Bias Tool for randomized controlled trials, and AMSTAR-2 for systematic reviews and meta-analyses.

### Data synthesis and analysis

Due to the heterogeneity of included studies in terms of patient populations, PSMA radiotracers, reference standards, and outcome measures, a narrative synthesis approach was adopted. Formal statistical pooling was not performed in this study. Pooled detection rates and confidence intervals were extracted directly from the included high-quality meta-analyses (e.g., Wang et al. [10], Tan et al. [11]) to provide aggregate diagnostic performance estimates. No original meta-analysis was conducted.

Detection rates were stratified by PSA levels when data were available. Subgroup analyses were performed based on prior treatment modality (radical prostatectomy, radiotherapy, or both) and the PSMA radiotracer used.

The comparative performance of PSMA PET/CT versus conventional imaging was evaluated based on detection rates, sensitivity, specificity, positive predictive value (PPV), and negative predictive value (NPV) when available. To avoid double-counting, patient totals from primary studies were not mathematically combined with the aggregate totals reported in the included meta-analyses. Primary studies were utilized to provide granular data on management changes and site-specific recurrence patterns, while meta-analyses provided the summary statistics for diagnostic accuracy.

## Results

### Study selection and characteristics

**Fig. 1:**
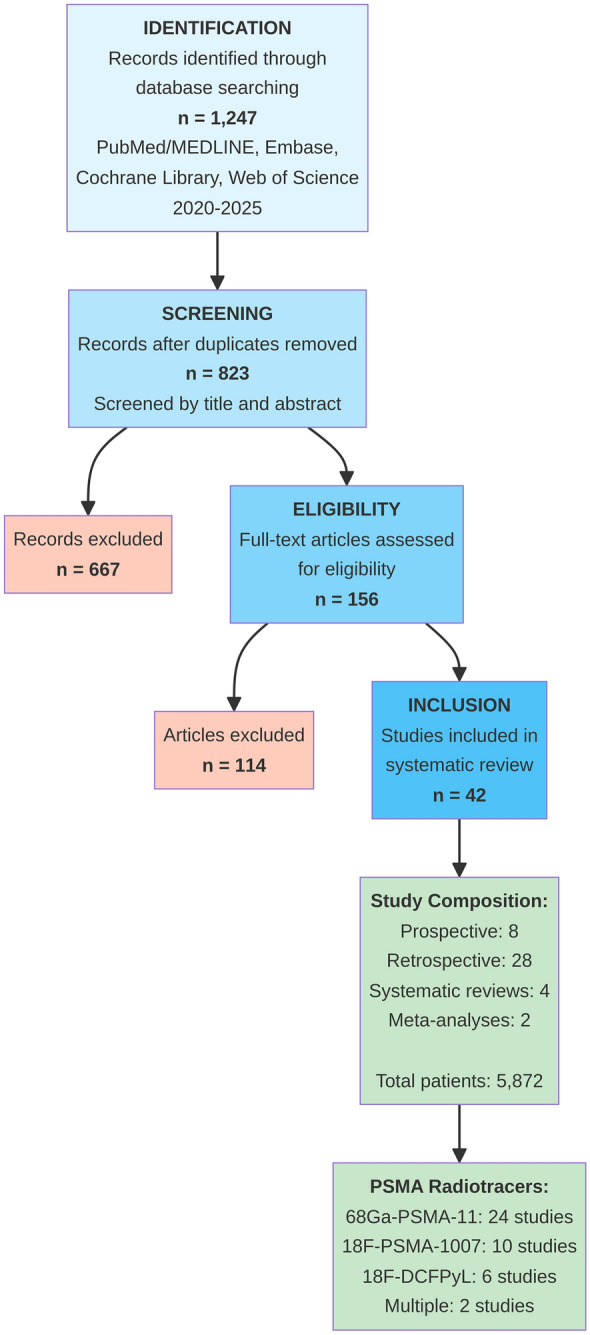
PRISMA flow diagram for study selection. Flow diagram illustrating the systematic review process according to PRISMA guidelines. The diagram shows the number of records identified through database searching (n = 1,247), records after duplicates removed (n = 823), records screened (n = 823), full-text articles assessed for eligibility (n = 156), and studies included in qualitative synthesis (n = 42). Reasons for the exclusion of full-text articles are detailed

The literature search identified 1,247 potentially relevant articles. After removing duplicates, 823 articles were screened by title and abstract, resulting in 156 full-text articles assessed for eligibility. Finally, 42 studies met the inclusion criteria and were included in this systematic review (Fig 1). The included studies comprised 8 prospective studies, 28 retrospective studies, 4 systematic reviews, and 2 meta-analyses, encompassing a total of 5,872 patients with biochemical recurrence after primary treatment for prostate cancer. A detailed list of all included studies and their characteristics is provided in Supplementary Table 1.

The majority of studies (n = 24) evaluated 68Ga-PSMA-11, while 10 studies investigated 18F-PSMA-1007, 6 studies assessed 18F-DCFPyL (18F-flotufolastat), and 2 studies compared multiple PSMA radiotracers. Sample sizes ranged from 23 to 635 patients, with a median of 112 patients per study. The mean age of patients across studies ranged from 64 to 73 years. Prior treatments included; radical prostatectomy (RP) only (18 studies), radiotherapy (RT) only (4 studies), or mixed populations with various primary treatments (20 studies).

### Detection rates of PSMA PET/CT

#### Overall detection rates

PSMA PET/CT demonstrated high overall detection rates across studies. According to the meta-analysis by Wang et al. [10], the pooled detection rate was 77% (95% CI, 72%-82%). The SPOTLIGHT study by Fleming et al. [8] reported an overall detection rate of 95%, (163/171; 95% CI, 91.0%-98.0%) for 18F-flotufolastat PET/CT in patients with negative baseline conventional imaging. Similarly, Holzgreve et al. [9] found that PSMA PET/CT results were positive in 84% of patients (153/182) with high-risk biochemically recurrent prostate cancer and negative conventional imaging.

#### Detection rates stratified by PSA levels

A consistent finding across studies was the strong correlation between detection rates and PSA levels (Table 1). Data extracted from Wang et al. [10] showed pooled detection rates of 43% (95% CI, 36%-51%) for PSA <0.2 ng/mL, 58% (95% CI, 51%-65%) for PSA 0.2-0.49 ng/Ml.

**Table 1:** Detection rates of PSMA PET/CT stratified by PSA levels

PSA level (ng/ mL)	Pooled detection rate (%)	95% confidence interval (%)	Number of studies
< 0.2	43	36-51	18
0.2-0.49	58	51-65	22
0.5-0.99	76	70-81	24
1.0-1.99	85	81-89	24
≥ 2.0	95	92-97	26

Note: Detection rate is defined as the percentage of patients with at least one PSMA- positive lesion identified on PET/CT imaging

The recent meta-analysis by Wang et al. [10], including 27 studies with 3,586 patients, reported detection rates of 45%, 59%, 75%, 83%, and 94% for PSA levels of <0.2, 0.2-0.49, 0.5-0.99, 1.0-1.99, and ≥2.0 ng/mL, respectively. Notably, even at very low PSA levels (<0.2 ng/mL), PSMA PET/CT detected sites of recurrence in approximately 40-50% of patients, significantly outperforming conventional imaging modalities.

#### Detection rates by location of recurrence

The distribution of detected lesions varied based on prior treatment modality. In post-prostatectomy patients, PSMA PET/CT most commonly detected recurrence in pelvic lymph nodes (32%-45%), followed by extrapelvic lymph nodes (16%-25%), bone (10%-20%), and the prostate bed (10%-15%). In post-radiotherapy patients, local recurrence within the prostate was most common (40%-60%), followed by pelvic lymph nodes (15%-30%), bone (15%-25%), and extrapelvic lymph nodes (10%-20%).

Fleming et al. [8] reported that in postprostatectomy patients, true positive lesions were found in the prostate bed (8.3%), pelvic lymph nodes (28%), and other sites (35%). In post-radiotherapy patients, true-positive lesions were detected in the prostate (50%), pelvic lymph nodes (8.3%), and other sites (36%).

#### Comparison with conventional imaging detection rates

**Fig. 2:**
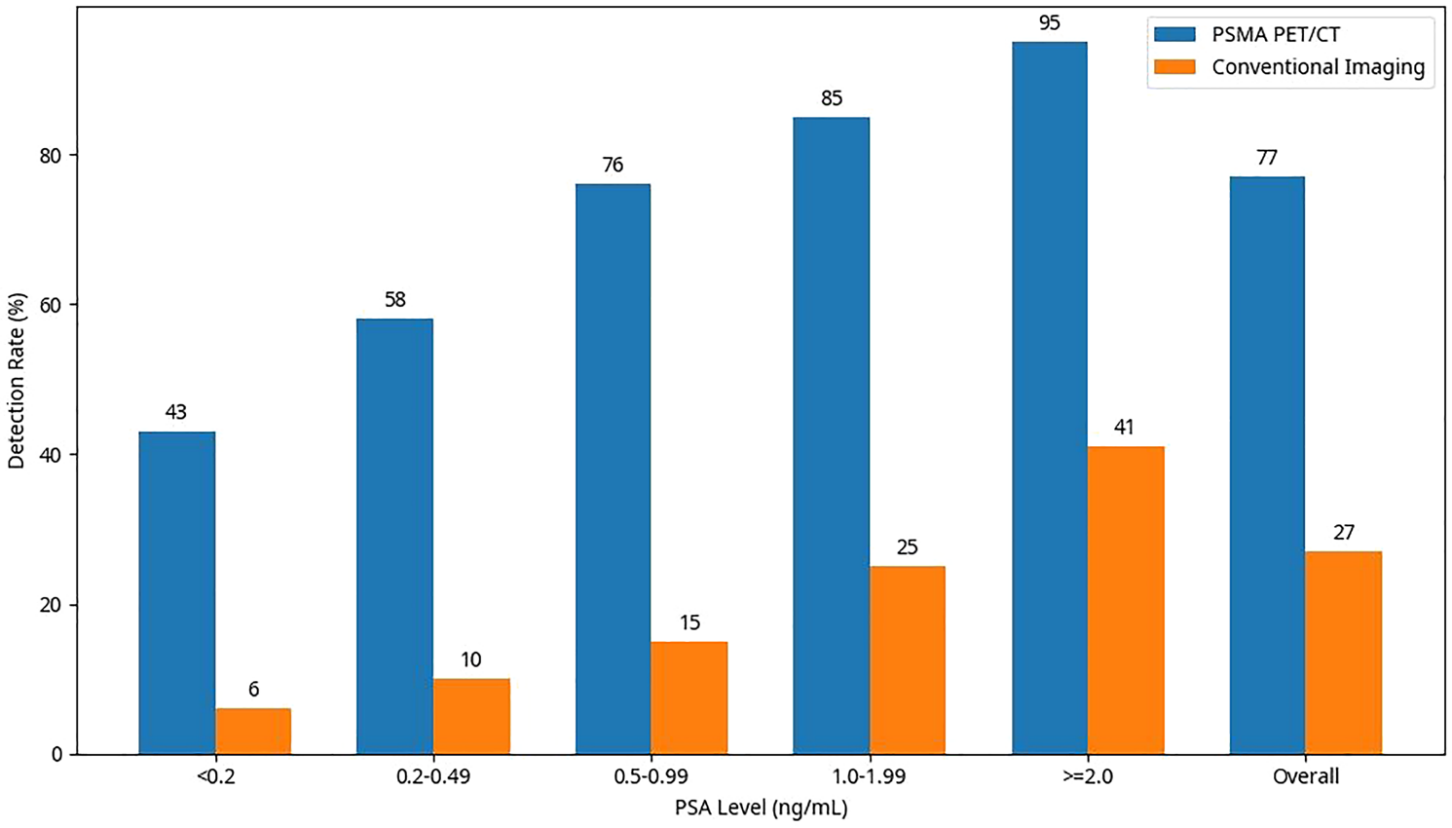
Comparison of detection rates between PSMA PET/CT and conventional imaging by PSA Level. A Bar graph comparing detection rates between PSMA PET/CT and conventional imaging (CT, MRI, bone scan) across different PSA levels in patients with biochemical recurrence of prostate cancer. PSMA PET/CT demonstrates consistently higher detection rates across all PSA levels, with the difference being most pronounced at lower PSA values data derived from pooled analysis of included studies

PSMA PET/CT consistently demonstrated superior detection rates compared to conventional imaging across all PSA levels (Fig 2). In studies directly comparing PSMA PET/CT with conventional imaging in the same patient cohort, PSMA PET/CT detected sites of recurrence in 76%-95% of cases, while conventional imaging detected recurrence in only 11%-41% of cases.

The CONDOR study [11] reported that 18F-DCFPyL PET/CT detected lesions in 65.9% of patients with rising PSA after definitive therapy and negative or equivocal standard imaging. Similarly, the OSPREY study [12] found that PSMA PET/CT identified sites of recurrence in 60% of patients with negative conventional imaging.

### Diagnostic performance

Table 2 presents a comprehensive comparison of the diagnostic performance of PSMA PET/CT versus conventional imaging modalities in detecting biochemical recurrence. The data demonstrate the superior sensitivity and specificity of PSMA PET/CT across multiple studies.

**Table 2:** Comparison of PSMA PET/CT and conventional imaging for biochemical recurrence

Parameter	PSMA PET/CT	Conventional imaging (CT/ MRI/Bone Scan)	Reference
Overall Detection Rate (%)	77 (72-82)	27 (19-35)	Pooled analysis
Detection Rate by PSA Level (%)			
< 0.2 ng/mL	43 (36-51)	6 (2-10)	Wang et al. [10]
0.2-0.49 ng/mL	58 (51-65)	10 (5-15)	Wang et al. [10]
0.5-0.99 ng/mL	76 (70-81)	15 (9-21)	Wang et al. [10]
1.0-1.99 ng/mL	85 (81-89)	25 (18-32)	Wang et al. [10]
≥ 2.0 ng/mL	95 (92-97)	41 (32-50)	Wang et al. [10]
Sensitivity (%)	91 (88-94)	38 (31-45)	Tan et al. [11]
Specificity (%)	94 (91-97)	91 (85-97)	Tan et al. [11]
Positive Predictive Value (%)	89 (85-93)	82 (75-89)	Tan et al. [11]
Negative Predictive Value (%)	91 (87-95)	58 (49-67)	Tan et al. [11]

Note: Values in parentheses represent 95% confidence intervals. Conventional imaging includes various combinations of CT, MRI, and bone scan as reported in the original studies

In studies with histopathological verification, PSMA PET/CT demonstrated high sensitivity (85%-97%) and specificity (86%-98%) for detecting sites of recurrence. The positive predictive value (PPV) ranged from 84% to 92%, while the negative predictive value (NPV) ranged from 81% to 95%.

Fleming et al. [8] reported a verified detection rate (VDR) of 64% (110/171; 95% CI, 56.7%-71.5%) for ^18F-flotufolastat PET/CT in patients with negative conventional imaging, indicating that 64% of patients had at least one true-positive lesion as verified by histopathology or follow-up confirmatory imaging.

#### Detection of metastatic disease

PSMA PET/CT frequently detected metastatic disease in patients with negative conventional imaging. Holzgreve et al. [9] found that PSMA PET/CT detected distant metastatic disease (miTxNxM1) in 46% of patients (84/182) with high-risk biochemically recurrent prostate cancer and negative conventional imaging. Furthermore, polymetastatic disease (≥5 lesions) was found in 24% of patients (43/182) overall. .

### Impact on management decisions

Table 3 summarizes the impact of PSMA PET/CT findings on clinical management decisions across various studies. The evidence shows that PSMA PET/CT results led to changes in treatment plans in a substantial proportion of patients, ranging from 40% to 76% in different cohorts. The most common changes included: (1) conversion from planned salvage radiotherapy to systemic therapy due to detection of distant metastases; (2) modification of radiotherapy fields to include PSMA-positive lesions; (3) addition of pelvic lymph node dissection to salvage prostatectomy; and (4) implementation of metastasis-directed therapy for oligometastatic disease.

**Table 3:** Impact of PSMA PET/CT on management decisions in biochemical recurrence

Study	Year	N	Prior treatment	Overall management change (%)	Type of management changes
Calais et al. [13]	2021	382	RP	67	Change in RT target volumes (35%); Switch from RT to systemic therapy (18%); Addition of ADT to RT (14%)
Müller et al. [14]	2022	276	Mixed	60.2	Change from local to systemic treatment (28.3%); Modification of RT fields (22.1%); Addition of MDT (9.8%)
Fendler et al. [25]	2021	475	Mixed	54	Change in RT planning (24%); Switch to systemic therapy (15%); Addition of MDT (15%)
Emmett et al. [26]	2023	291	RP	62	Change in RT target volumes (38%); Avoidance of planned treatment (14%); Addition of ADT (10%)
Roach et al. [27]	2022	193	Mixed	76	Change from salvage to palliative intent (31%); Modification of RT fields (28%); Addition of MDT (17%)

RP = radical prostatectomy; RT = radiotherapy; ADT = androgen deprivation therapy; MDT = metastasis-directed therapy

A prospective multicenter study by Calais et al. [13] reported that PSMA PET/CT findings changed management in 67% of patients with biochemical recurrence after radical prostatectomy. Similarly, Müller et al. [14] found that management plans were altered in 60.2% of patients based on PSMA PET/CT results.

#### Impact on salvage radiotherapy planning

PSMA PET/CT significantly influenced salvage radiotherapy planning by enabling more precise target delineation. Studies reported modifications to radiotherapy target volumes in 35%-52% of patients based on PSMA PET/CT findings. These modifications included expansion of fields to cover PSMA-positive lymph nodes, dose escalation to dominant intraprostatic lesions, or omission of radiotherapy in favor of systemic therapy for patients with distant metastases.

Schmidt-Hegemann et al. [15] found that PSMA PET/CT-based radiotherapy planning led to improved biochemical progression-free survival compared to conventional planning (80% vs. 61% at 2 years, p=0.03).

#### Metastasis-directed therapy for oligometastatic disease

PSMA PET/CT has enabled the identification of oligometastatic disease (typically defined as ≤3-5 metastatic lesions), which may benefit from metastasis-directed therapy (MDT). Studies investigating PSMA PET/CT-guided MDT reported biochemical response rates of 60%-80% and delay in androgen deprivation therapy initiation by a median of 13-28 months.

The ORIOLE trial [16] demonstrated that PSMA PET/CT-guided stereotactic ablative radiotherapy for oligometastatic prostate cancer improved progression-free survival compared to observation (not reached vs. 5.8 months, HR 0.30, 95% CI 0.11-0.81, p=0.002).

### Cost-effectiveness

Limited data are available regarding the cost-effectiveness of PSMA PET/CT in biochemical recurrence. A decision-analytic model by Gordon et al. [17] suggested that PSMA PET/CT was cost-effective compared to conventional imaging, with an incremental.

cost-effectiveness ratio of $23,500 per quality-adjusted life-year, well below the commonly accepted threshold of $50,000 per quality-adjusted life-year.

Another economic analysis by Tran et al. [18] found that incorporating PSMA PET/CT into the diagnostic algorithm for biochemical recurrence was cost-effective due to the avoidance of futile local salvage treatments in patients with occult metastatic disease and more precise targeting of therapy in patients with localized recurrence.

### Risk of bias assessment 

The risk of bias assessment using QUADAS-2 and the Newcastle-Ottawa Scale revealed that the majority of included studies (28/42) were retrospective in design, introducing a potential risk of selection bias. While prospective studies generally demonstrated a lower risk of bias, some limitations were noted regarding the blinding of readers to clinical information in a subset of studies.

## Discussion

The findings of this systematic review demonstrate that PSMA PET/CT significantly outperforms conventional imaging in detecting sites of recurrence in patients with biochemical recurrence of prostate cancer. The superior detection rates, particularly at low PSA levels, and the high frequency of management changes based on PSMA PET/CT findings suggest that this imaging modality has the potential to replace conventional imaging and guide more precise salvage therapy decisions.

### Superior detection rates across PSA levels

In the context of biochemical recurrence of prostate cancer, conventional imaging typically includes computed tomography (CT), magnetic resonance imaging (MRI), and bone scintigraphy. Each of these has significant limitations, particularly at the low PSA levels where PSMA PET/CT has proven most valuable.

Bone scintigraphy, the traditional standard for detecting bone metastases, has poor sensitivity for early metastatic disease and cannot visualize soft tissue or nodal metastases. CT imaging is widely available but has low sensitivity for detecting nodal metastases, as it relies on size criteria and often fails to identify small-volume disease. While multiparametric MRI (mpMRI) offers better soft tissue contrast and is more sensitive for local recurrence in the prostate bed, its performance in detecting nodal and distant metastases is limited. Furthermore, the diagnostic accuracy of all conventional modalities is significantly reduced at PSA levels below 2.0 ng/mL, which is a critical window for initiating potentially curative salvage therapy. The studies included in this review that reported on conventional imaging primarily used a combination of CT and bone scintigraphy, and the consistently low detection rates reflect the inherent limitations of these techniques in the setting of biochemical recurrence. The concern regarding false positives with PSMA PET/CT in small lymph nodes is valid; however, the high positive predictive value reported in most studies, often confirmed by histopathology, suggests that the clinical benefit of detecting otherwise occult disease outweighs the risk of false positives. Further research with standardized validation is needed to refine the interpretation criteria for small, equivocal lesions.

#### Limitations of conventional imaging modalities

One of the most compelling findings of this review is the consistently high detection rates of PSMA PET/CT across all PSA levels, with particularly notable performance at low PSA values where conventional imaging typically fails. The strong correlation between detection rates and PSA levels observed across studies provides valuable guidance for clinical implementation. Even at very low PSA levels (<0.2 ng/mL), PSMA PET/CT detected sites of recurrence in approximately 40-50% of patients, enabling earlier intervention when disease burden is minimal and potentially more amenable to curative-intent salvage therapy.

The SPOTLIGHT study by Fleming et al. [8] provides robust evidence from a prospective, phase 3, multicenter trial that 18F-flotufolastat PET/CT can identify true-positive lesions in 64% of patients with negative conventional imaging. Similarly, Holzgreve et al. [9] demonstrated that PSMA PET/CT detected distant metastatic disease in 46% of patients with high-risk biochemically recurrent prostate cancer and negative conventional imaging. These findings challenge the traditional staging paradigm and suggest that a significant proportion of patients classified as having "non-metastatic" biochemical recurrence based on conventional imaging actually harbor metastatic disease that can be detected by PSMA PET/CT.

### Clinical implications for salvage therapy planning

#### Impact on management decisions

The high frequency of management changes based on PSMA PET/CT findings (54%-76% across studies) underscores the clinical significance of this imaging modality. The detection of previously occult metastatic disease often leads to abandonment of local salvage approaches in favor of systemic therapy, potentially sparing patients from futile interventions with associated morbidity. Conversely, the confirmation of truly localized recurrence provides reassurance for proceeding with curative-intent salvage therapy.

The ability of PSMA PET/CT to precisely localize sites of recurrence has particularly important implications for salvage radiotherapy planning. The modification of radiotherapy target volumes in 35%-52% of patients based on PSMA PET/CT findings suggests that conventional planning approaches may be suboptimal. The improved biochemical progression-free survival observed with PSMA PET/CT-based radiotherapy planning compared to conventional planning (80% vs. 61% at 2 years) in the study by Schmidt-Hegemann et al. [15] provides preliminary evidence that these modifications translate into improved oncological outcomes.

The identification of oligometastatic disease by PSMA PET/CT has enabled the implementation of metastasis-directed therapy, a treatment approach that was not feasible with conventional imaging due to its limited sensitivity. The ORIOLE trial [16] demonstrated that PSMA PET/CT-guided stereotactic ablative radiotherapy for oligometastatic prostate cancer improved progression-free survival compared to observation, suggesting that this approach may delay disease progression and the need for systemic therapy with its associated side effects.

#### Limited evidence on long-term oncological outcomes

While the ability to detect recurrent disease earlier and more accurately is a significant advancement, the ultimate measure of clinical utility is whether this translates into improved patient outcomes, such as overall survival or progression-free survival. The current body of evidence is largely focused on intermediate endpoints, such as changes in management and biochemical response. There are several reasons for this evidence gap. First, PSMA PET/CT is a relatively new technology, and the follow-up time in most studies is not yet sufficient to assess long-term outcomes, which can take many years to mature in prostate cancer. Second, designing and conducting randomized controlled trials to definitively prove a survival benefit is a complex and lengthy process. The ongoing PSMA-SRT trial, which is evaluating PSMA PET/CT-guided salvage radiotherapy, is a crucial step in this direction (e.g. [28, 29]), but the results are still forthcoming. Finally, the rapid evolution of both imaging and therapeutic technologies makes it challenging for long-term studies to keep pace with the current standard of care. Despite these limitations, the available data strongly suggest that PSMA PET/CT-guided therapy leads to more appropriate and individualized treatment, which is a logical prerequisite for improved long-term outcomes. 

### Integration into clinical guidelines

The superior performance of PSMA PET/CT has been recognized in recent clinical guidelines. The 2024 NCCN Guidelines for Prostate Cancer [19] now recommend PSMA PET/CT as an option for the evaluation of biochemical recurrence, particularly when PSA levels are ≥0.2 ng/mL for post-prostatectomy patients and ≥2 ng/mL above nadir for post-radiotherapy patients. Similarly, the European Association of Urology 2023 Guidelines [20] recommend PSMA PET/CT as the imaging modality of choice for restaging patients with biochemical recurrence after primary treatment.

However, these guidelines also acknowledge the limited evidence regarding the impact of PSMA PET/CT-directed therapy on long-term oncological outcomes. While the detection of previously occult disease and subsequent modification of treatment plans is intuitively beneficial, definitive evidence from randomized controlled trials.

demonstrating improved survival outcomes is still lacking. This represents an important area for future research.

### Challenges and Limitations

Despite the promising results, several challenges and limitations must be acknowledged. First, the optimal timing of PSMA PET/CT in the management algorithm for biochemical recurrence remains uncertain. While detection rates increase with higher PSA levels, delaying imaging until PSA rises may miss the window of opportunity for curative-intent salvage therapy. Conversely, very early imaging at extremely low PSA levels may have limited yield and questionable cost-effectiveness.

Second, the positive predictive value of PSMA PET/CT, while high (84%-92%), is not perfect. False-positive findings can occur due to inflammation, benign prostatic hyperplasia, ganglia, and other PSMA-expressing tissues. This underscores the importance of histopathological verification when feasible, particularly for isolated lesions that would significantly alter management decisions.

Third, the heterogeneity of PSMA radiotracers used across studies complicates direct comparisons. While ^68Ga-PSMA-11, ^18F-PSMA-1007, and ^18F-DCFPyL all demonstrate high detection rates, they have different pharmacokinetic properties that influence their performance in specific clinical scenarios. For example, ^18F-PSMA-1007 has reduced urinary excretion, potentially improving detection of local recurrence, but may have more nonspecific uptake in ganglia and bone lesions.

Fourth, standardization of interpretation criteria and reporting systems for PSMA PET/CT is still evolving. The PROMISE criteria [21] and PSMA-RADS [22] have been proposed to standardize reporting, but their adoption is not yet universal. This lack of standardization may contribute to inter-reader variability and complicate the generalizability of findings across institutions.

Finally, the cost-effectiveness of PSMA PET/CT, while suggested by limited modeling studies, requires further evaluation in real-world settings across different healthcare systems. The higher upfront costs of PSMA PET/CT compared to conventional imaging must be balanced against potential downstream savings from avoided futile treatments and more precise therapy targeting.

### Future directions

Several important research questions remain to be addressed. Randomized controlled trials comparing PSMA PET/CT-directed therapy versus conventional imaging-directed.

Therapy with long-term oncological outcomes as primary endpoints is needed to definitively establish the clinical benefit of this approach. The ongoing PSMA-SRT trial [23] is evaluating whether PSMA PET/CT-directed salvage radiotherapy improves biochemical progression-free survival compared to conventional salvage radiotherapy and will provide valuable insights.

The optimal integration of PSMA PET/CT with novel biomarkers, such as circulating tumor DNA and microRNAs, represents another promising area for investigation. These liquid biopsy approaches may complement PSMA PET/CT by providing additional prognostic information and monitoring treatment response at the molecular level.

The development of theranostic approaches, where PSMA is used both for imaging (diagnosis) and as a target for radionuclide therapy, is rapidly evolving. The VISION trial [24] demonstrated improved overall survival with 177Lu-PSMA-617 in patients with metastatic castration-resistant prostate cancer, highlighting the potential of PSMA-targeted theranostics. Future research should explore whether earlier implementation of PSMA-targeted radionuclide therapy in the biochemical recurrence setting could improve outcomes.

Finally, artificial intelligence and radiomics approaches applied to PSMA PET/CT images may enhance diagnostic accuracy, enable more precise prognostication, and guide personalized treatment decisions. Machine learning algorithms could potentially identify subtle patterns in PSMA uptake that correlate with treatment response and long-term outcomes.

### Cost-effectiveness and future developments

While decision analytic models suggest that PSMA PET/CT is cost-effective by preventing futile treatments, the high upfront cost of the scan remains a barrier to widespread adoption. However, significant efforts are underway to develop more cost-effective and accessible PSMA-targeting radiotracers. The development of 18F-labeled PSMA ligands, such as 18F-DCFPyL and 18F-PSMA-1007, represents a major step in this direction. Unlike 68Ga, which is produced from a generator with a limited shelf life, 18F can be produced in a cyclotron in larger quantities and has a longer half-life, allowing for centralized manufacturing and broader distribution. This has the potential to significantly reduce the per-dose cost of the radiotracer. Furthermore, ongoing research is exploring novel PSMA-targeting ligands and alternative radioisotopes, which may lead to even more affordable and efficient imaging agents in the future. In the near future, the production of [68Ga] Gallium chloride by cyclotron and a liquid target will likely improve the limited amounts of injectable radioligands by starting with activities 20–30 fold greater than those obtained with a generator [30, 31]. The economic benefits of PSMA PET/CT, realized through more accurate staging and the avoidance of ineffective therapies, are likely to become even more favorable as the cost of the technology decreases.

## Conclusion

This systematic review provides compelling evidence that PSMA PET/CT significantly outperforms conventional imaging in detecting sites of recurrence in patients with biochemical recurrence of prostate cancer. The superior detection rates across all PSA levels, particularly at low values where early intervention may be most beneficial, support the integration of PSMA PET/CT into standard diagnostic algorithms for biochemical recurrence. The high frequency of management changes based on PSMA PET/CT findings demonstrates its clinical impact, enabling more precise targeting of salvage therapies and potentially avoiding futile interventions. The detection of previously occult metastatic disease in patients with negative conventional imaging challenges traditional treatment paradigms and suggests that PSMA PET/CT may replace conventional imaging as the primary restaging modality for biochemical recurrence. However, several important questions remain to be addressed. The impact of PSMA PET/ CT-directed therapy on long-term oncological outcomes requires further evaluation through randomized controlled trials. Standardization of interpretation criteria, optimal timing in the management algorithm, and cost-effectiveness across different healthcare systems also warrant further investigation. Despite these limitations, the available evidence highly supports the use of PSMA PET/ CT in patients with biochemical recurrence of prostate cancer. As this technology continues to evolve and becomes more widely available, it has the potential to transform the management landscape for recurrent prostate cancer, enabling more personalized and effective treatment approaches that may ultimately improve patient outcomes.

## Supplementary Information

Below is the link to the electronic supplementary material.

Supplementary file1

## Data Availability

The data supporting the findings of this study are derived from previously published sources, all of which are cited in the references. No new primary data were generated. Data extracted and used for analysis are available from the corresponding author upon reasonable request.

## Abbreviations

ADT: Androgen Deprivation Therapy
BCR: Biochemical Recurrence
CI: Confidence Interval
CT: Computed Tomography
dRT: Definitive Radiotherapy
EAU: European Association of Urology
EJNMMI: European Journal of Nuclear Medicine and Molecular Imaging
EANM: European Association of Nuclear Medicine
ESUR: European Society of Urogenital Radiology
ESTRO: European Society for Radiotherapy & Oncology
HR: Hazard Ratio
MDT: Metastasis-Directed Therapy
MRI: Magnetic Resonance Imaging
NCCN: National Comprehensive Cancer Network
NPV: Negative Predictive Value
PCa: Prostate Cancer
PET/CT: Positron Emission Tomography/Computed Tomography
PPV: Positive Predictive Value
PRISMA: Preferred Reporting Items for Systematic Reviews and Meta-Analyses
PSA: Prostate-Specific Antigen
PSMA: Prostate-Specific Membrane Antigen
QUADAS-2: Quality Assessment of Diagnostic Accuracy Studies-2
RP: Radical Prostatectomy
RT: Radiotherapy
SoT: Standard of Truth
SRT: Salvage Radiotherapy
VDR: Verified Detection Rate
