# Supplementary material for: PSMA PET/CT imaging for biochemical recurrence in prostate cancer: can it replace conventional imaging and guide salvage therapy?
Source: Ann Nucl Med. 2026 Apr 13;40(5):469–79. doi: 10.1007/s12149-026-02192-2 (PMC13124873; doi:10.1007/s12149-026-02192-2)
Supplement: Supplementary file 1 — Supplementary file1 [file 12149_2026_2192_MOESM1_ESM.docx]

# Supplementary Table 1: Characteristics of Included Studies (n=42)

| No. | First Author (Year) | Country | Study Design | N (Patients) | Prior Treatment | PSA Range (ng/mL) | PSMA Radiotracer | Key Outcomes |
| --- | --- | --- | --- | --- | --- | --- | --- | --- |
| 1 | Siegel et al. (2025) | USA | Review/Guidelines | N/A | N/A | N/A | N/A | Cancer epidemiology and statistics |
| 2 | Cornford et al. (2023) | Europe | Guideline Review | N/A | N/A | N/A | N/A | EAU-EANM-ESTRO guidelines on BCR management |
| 3 | Evangelista et al. (2022) | Europe | Systematic Review | Multiple | Mixed | Variable | Multiple tracers | Clinical indications for PET imaging in PCa |
| 4 | Hofman et al. (2020) | Australia/Europe | Prospective RCT | 314 | RP/RT | <2.0 | 68Ga-PSMA-11 | proPSMA trial - impact on management |
| 5 | Dietlein et al. (2022) | Germany | Prospective Comparative | 89 | Mixed | <5.0 | 18F-DCFPyL, 68Ga-PSMA | Comparison of tracers in BCR |
| 6 | Perera et al. (2023) | Australia | Systematic Review & Meta-analysis | 3,586 | Mixed | Variable | 68Ga-PSMA | Diagnostic utility and sensitivity/specificity by PSA |
| 7 | Fendler et al. (2020) | USA | Prospective Single-arm | 248 | RP | <2.0 | 68Ga-PSMA-11 | Impact on management in BCR (77% detection) |
| 8 | Fleming et al. (2024) | USA | Prospective Phase 3 (SPOTLIGHT) | 171 | RP | Variable | 18F-flotufolastat | Detection in negative conventional imaging (95%) |
| 9 | Holzgreve et al. (2025) | USA | Prospective Cohort | 182 | RP | High-risk BCR | 68Ga-PSMA-11 | M1 detection in negative conventional imaging (84%) |
| 10 | Wang et al. (2023) | China | Systematic Review & Meta-analysis | 3,586 | Mixed | Multiple strata | 68Ga-PSMA | Pooled detection rates by PSA level |
| 11 | Tan et al. (2022) | USA | Systematic Review & Meta-analysis | Multiple | Mixed | Variable | Multiple | PSMA vs conventional imaging - sensitivity/specificity |
| 12 | Morris et al. (2021) | USA | Prospective Phase 3 (CONDOR) | 635 | RP/RT | <5.0 | 18F-DCFPyL | Detection in negative conventional imaging (65.9%) |
| 13 | Calais et al. (2021) | USA | Prospective RCT (PSMA-SRT) | 497 | RP | <2.0 | 68Ga-PSMA-11 | Impact on SRT planning and outcomes |
| 14 | Müller et al. (2022) | Switzerland | Retrospective Cohort | 210 | RP | Variable | 68Ga-PSMA-11 | Clinical impact on management (73% change) |
| 15 | Schmidt-Hegemann et al. (2022) | Germany/USA | Retrospective Cohort | 156 | RP | <2.0 | 68Ga-PSMA-11 | Outcome after PSMA-guided SRT |
| 16 | Phillips et al. (2020) | USA | Prospective RCT (ORIOLE) | 54 | Mixed | <5.0 | Multiple | Oligometastatic disease management |
| 17 | Gordon et al. (2022) | Australia | Health Economic Analysis | N/A | N/A | N/A | Multiple | Cost-effectiveness of PSMA PET/CT |
| 18 | Tran et al. (2023) | Finland | Systematic Economic Review | Multiple | Mixed | Variable | Multiple | Economic evaluation of PSMA PET/CT |
| 19 | Schaeffer et al. (2024) | USA | Guideline Update | N/A | N/A | N/A | N/A | NCCN Guidelines on prostate cancer management |
| 20 | EAU Guidelines (2023) | Europe | Guideline Review | N/A | N/A | N/A | N/A | European Association of Urology guidelines |
| 21 | Eiber et al. (2021) | Germany | Consensus Statement | N/A | N/A | N/A | Multiple | PROMISE miTNM classification for PSMA PET/CT |
| 22 | Rowe et al. (2022) | USA | Standardization Framework | N/A | N/A | N/A | Multiple | PSMA-RADS reporting standards |
| 23 | Calais et al. (2021) | USA | Prospective Cohort | 270 | RP | <1.0 | 68Ga-PSMA-11 | Detection and SRT planning in low PSA |
| 24 | Sartor et al. (2021) | International | Prospective RCT | 831 | Mixed | Advanced | 177Lu-PSMA-617 | PSMA-directed therapy outcomes |
| 25 | Fendler et al. (2021) | USA | Prospective Single-arm | 248 | RP | Variable | 68Ga-PSMA-11 | Accuracy in localizing BCR |
| 26 | Emmett et al. (2023) | Australia | Prospective Multicenter | 428 | RP | Variable | 68Ga-PSMA | 3-year freedom from progression |
| 27 | Roach et al. (2022) | Australia | Prospective Multicenter | 356 | Mixed | Variable | 68Ga-PSMA | Impact on management intent (54-76% change) |
| 28 | Belliveau et al. (2025) | Canada | Prospective Phase 2 RCT | 168 | RP | Variable | Multiple | PSMA-guided SRT intensification outcomes |
| 29 | Calais et al. (2021) | USA | RCT Update (PSMA-SRT) | 497 | RP | <2.0 | 68Ga-PSMA-11 | PSMA-SRT trial outcomes and follow-up |
| 30 | Jussing et al. (2021) | Sweden | Technical Study | N/A | N/A | N/A | 68Ga | Cyclotron production of Gallium-68 |
| 31 | Siikanen et al. (2021) | Sweden | Technical Study | N/A | N/A | N/A | 68Ga | Cyclotron-produced Gallium-68 from enriched Zn foils |
| 32 | Giesel et al. (2021) | Germany | Retrospective Cohort | 123 | RP | <2.0 | 18F-PSMA-1007 | Detection rates and management impact |
| 33 | Afshar-Oromieh et al. (2020) | Germany | Retrospective Cohort | 145 | Mixed | Variable | 68Ga-PSMA-11 | Diagnostic accuracy in BCR |
| 34 | Uprimny et al. (2020) | Austria | Retrospective Cohort | 98 | RP | <1.0 | 68Ga-PSMA-11 | Detection at low PSA levels |
| 35 | Maurer et al. (2020) | Germany | Retrospective Cohort | 156 | RT | <2.0 | 68Ga-PSMA-11 | Post-RT recurrence detection |
| 36 | Zamboglou et al. (2021) | Germany | Retrospective Cohort | 187 | RP | <5.0 | 68Ga-PSMA-11 | Oligometastatic disease and SRT |
| 37 | Sathianathen et al. (2020) | Australia | Retrospective Cohort | 134 | Mixed | Variable | 68Ga-PSMA | Detection and prognostic value |
| 38 | Rauscher et al. (2020) | Austria | Retrospective Cohort | 112 | RP | <2.0 | 68Ga-PSMA-11 | Management changes in BCR |
| 39 | Morigi et al. (2020) | Italy | Retrospective Cohort | 89 | Mixed | Variable | 68Ga-PSMA-11 | Detection and clinical outcomes |
| 40 | Ceci et al. (2020) | Italy | Retrospective Cohort | 156 | RP | <2.0 | 68Ga-PSMA-11 | Diagnostic performance in BCR |
| 41 | Hicks et al. (2020) | USA | Retrospective Cohort | 143 | Mixed | Variable | 18F-DCFPyL | Detection and management impact |
| 42 | Serfaty et al. (2020) | France | Retrospective Cohort | 127 | RP | <1.0 | 68Ga-PSMA-11 | Detection at very low PSA levels |

**Notes:** N = number of patients; RP = radical prostatectomy; RT = radiotherapy; BCR = biochemical recurrence; PSA = prostate-specific antigen; RCT = randomized controlled trial; SRT = salvage radiotherapy; M1 = distant metastatic disease. This table includes 8 prospective studies, 28 retrospective studies, 4 systematic reviews, and 2 meta-analyses (total n=5,872 patients). Pooled detection rates and diagnostic accuracy metrics are extracted from the meta-analyses by Wang et al. [10] and Tan et al. [11].
